# Resolving Species Level Changes in a Representative Soil Bacterial Community Using Microfluidic Quantitative PCR

**DOI:** 10.3389/fmicb.2017.02017

**Published:** 2017-10-25

**Authors:** Hannah Kleyer, Robin Tecon, Dani Or

**Affiliations:** Soil and Terrestrial Environmental Physics, Department of Environmental Systems Science, ETH Zürich, Zurich, Switzerland

**Keywords:** synthetic community, real-time PCR, microbial ecology, absolute quantification, community assembly, soil bacterial community, Fluidigm, porous media

## Abstract

Rapid advances in genome sequencing technologies enable determination of relative bacterial abundances and community composition, yet, changes at the species level remain difficult to detect despite importance for certain ecological inferences. We present a method for extraction and direct quantification of species composition of a predefined multispecies bacterial community using microfluidic-based quantitative real-time PCR (qPCR). We employ a nested PCR approach based on universal 16S rRNA gene pre-amplification followed by detection and quantification of absolute abundance of bacterial species using microfluidic array of parallel singleplex qPCR reactions. Present microfluidic qPCR supports 2,304 simultaneous reactions on a single chip, while automatic distribution of samples and reactants minimizes pipetting errors and technical variations. The utility of the method is illustrated using a synthetic soil bacterial community grown in two contrasting environments – sand microcosms and batch cultures. The protocol entails extraction of total nucleic acid, preparation of genomic DNA, and steps for qPCR assessment of bacterial community composition. This method provides specific and sensitive quantification of bacterial species requiring only 2 ng of community DNA. Optimized extraction protocol and preamplification step allow for rapid, quantitative, and simultaneous detection of candidate species with high throughput. The proposed method offers a simple and accurate alternative to present sequencing methods especially when absolute values of species abundance are required. Quantification of changes at the species level contributes to the mechanistic understanding of the roles of particular species in a bacterial community functioning.

## Introduction

**FIGURE 1:**
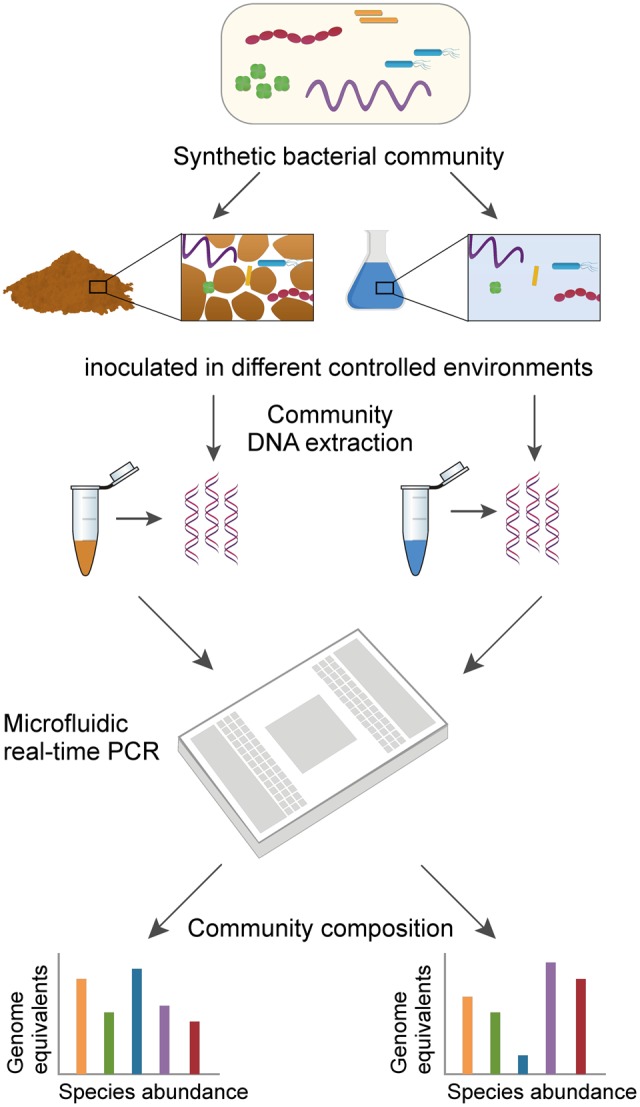
Workflow to assess the composition of a multispecies bacterial community using high-throughput qPCR. Schematic representation shows the main steps of the method. A synthetic bacterial community (i.e., an artificial assembly of well-characterized species) is incubated under various controlled conditions that result in different community compositions over time. Following community DNA extraction from various environments, the abundance of 16S rRNA genes from each individual species is quantified on a single Fluidigm 48 × 48 Dynamic Array. In addition to relative abundance, absolute abundance of species can be expressed as equivalent genome copy numbers back-calculated for each species using standard curves run on the same array.

The study of microbial communities in natural environments is hindered by inherent biological complexity and heterogeneous local abiotic conditions that obscure hypothesis testing and mechanistic understanding (Prosser et al., 2007). In recent years, the field of synthetic ecology has emerged as a promising framework for quantitative study of community assembly and functioning (Yu et al., 2016). Synthetic microbial ecology uses well-characterized microbial species with a well-defined genetic background as building blocks for artificial ecological systems studied and manipulated in controlled environments (Song et al., 2014; Dolinsek et al., 2016; Widder et al., 2016). The relative simplicity of synthetic systems facilitates the investigation and identification of mechanistic processes. The dynamics of such microbial community assembly and its stability and function under controlled conditions have been the focus of recent studies (Jousset et al., 2013; Bodenhausen et al., 2014; Yu et al., 2016). For many studies, detailed assessment of changes in microbial community composition is required. A range of techniques has been used for the quantification based on the detection of the ribosomal RNA genes, such as denaturing gradient gel electrophoresis (DGGE) (Muyzer and Smalla, 1998), automated ribosomal intergenic spacer analysis (ARISA) (Bodenhausen et al., 2014), and terminal restriction fragment length polymorphism (T-RFLP) analysis (Angel et al., 2010). More recently, these techniques have been replaced by high-throughput 16S rRNA amplicon sequencing due to the widespread availability and decrease in analysis costs (Props et al., 2017). However, while such fingerprinting and sequencing methods provide a general overview of the relative abundance and activity within a microbial community, they do not provide information on absolute abundances but express relative abundance or proportions of certain operational taxonomic units (OTUs) (Widder et al., 2016). A limitation of such methods is that changes in the relative abundance of OTUs contain limited information on total bacteria number or on bacterial biomass in a sample (Smets et al., 2016; Stämmler et al., 2016). Relative shifts (gains or losses) in OTUs numbers may result in similar changes in relative abundance, yet, such changes may reflect different ecological adaptations. Additionally, the lack of information on increase or decrease in an individual species (or OTU) abundance among different samples has been shown to introduce a bias in the evaluation of actual ratios that could be avoided by included total abundance counts (Props et al., 2017). Quantitative real-time PCR (qPCR) is widely used to determine values of absolute abundance, due to its robustness, sensitivity, and high reproducibility in recording quantitative changes of phylogenetic and functional gene markers across spatial and temporal scales (Smith and Osborn, 2009). Relatively recently, the development of microfluidic qPCR has permitted a more than 10-fold increase in the number of qPCR reactions that could be simultaneously run on a single platform (Spurgeon et al., 2008), with up to 9,216 parallel reactions performed on the densest microfluidic chips (Ishii et al., 2013). Here we report a microfluidic qPCR-based method to assess relative and absolute abundance of bacterial species in a model synthetic community, and to add definitiveness and improve research capabilities in the field of synthetic (and natural) microbial ecology (workflow illustrated in **Figure 1**). The purpose of this study was to implement a method for simultaneous detection and quantification of absolute abundance of community composition down to species level to record responses to (variations of) experimental or environmental conditions. This method is particularly well suited for hypothesis testing on the assembly of synthetic microbial communities (i.e., with *a priori* knowledge on community members).

To illustrate the method, we inoculated a representative bacterial community (**Table 1**) in two contrasting model environments, sand microcosms representing a spatially complex and unsaturated porous environment such as found in most terrestrial habitats and, in contrast, shaken batch cultures (containing sand) representing well-mixed, unstructured habitats. We assumed that incubation in the two contrasting environments would result in distinct community compositions that could be revealed by microfluidic qPCR analysis. The microcosms consisted of a layer of quartz sand placed on a porous ceramic disk and hydrated with liquid growth medium. Ceramic surfaces have been used previously as a porous surface model for the growth of soil bacteria under controlled hydration and nutrient conditions (Dechesne et al., 2008, 2010). We applied a controlled suction (matric potential head) to the porous plate in order to set a partially saturated condition in the sand phase. The same liquid growth medium was used in the shaken batch cultures which represented well-mixed, unstructured habitats. We compared aspects of extraction from sand phase and liquid media and response to a structured environment, and availability of water in terms of relative and absolute species abundances. The proposed qPCR method to assess bacterial community composition on one microfluidic qPCR chip increases information throughput by raising the number of parallel reactions in comparison with conventional real-time PCR in microtiter plates. In this study, the microfluidic chip enabled us to run 2,304 parallel reactions with up to 48 DNA samples and 48 distinct PCR assays. The integrated fluidic circuits automatically distribute and combine each DNA sample and each assay (Master Mix and individual primers) in the individual reaction microchambers on the chip. Pipetting errors are thus minimized, and variation among technical replicates became negligible. Potential applications may include the need for tracking and quantifying specific functional groups or indicator species relevant for ecosystem functioning and services (Torsvik and Øvreås, 2002), or the monitoring of emerging or reemerging specific pathogens (McCabe et al., 1999). With *a priori* knowledge on the target bacterial groups or species even uncultured strains could be detected and quantified, the proposed workflow allows to adopt the assay to different target groups or species depending on the research question.

**Table 1:** Bacterial strains used in this study.

Species	Strain	Gram	Phylum	Origin	Cells	Motile	Sporulating
*Arthrobacter chlorophenolicus*	A6^T^	+	Actinobacteria	Soil	Rods	-	-
*Bacillus subtilis*	168 trp+	+	Firmicutes	Soil	Rods	+	+
*Burkholderia xenovorans*	LB400^T^	-	β-Proteobacteria	Rhizosphere	Rods	+	-
*Escherichia coli*	MG1655	-	γ-Proteobacteria	Human gut	Rods	+	-
*Micrococcus luteus*	DSM20030^T^	+	Actinobacteria	Soil	Cocci	-	-
*Paenibacillus sabinae*	T27^T^	+	Firmicutes	Rhizosphere	Rods	+	+
*Pseudomonas protegens*	CHA0^T^	-	γ-Proteobacteria	Soil	Rods	+	-
*Pseudomonas stutzeri*	CMT.9.A	-	γ-Proteobacteria	Rhizosphere	Rods	+	-
*Rhizobium etli*	CFN 42^T^	-	α-Proteobacteria	Rhizosphere	Rods	+	-
*Streptomyces violaceoruber*	A3(2)	+	Actinobacteria	Soil	Filaments	-	+
*Xanthobacter autotrophicus*	7C^T^	-	α-Proteobacteria	Soil	Rods	-	-

*^T^Type strain.*

## Materials and Methods

### Bacterial Strains and Culture Conditions

The bacterial strains selected for this study were assembled as a synthetic community representing common bacterial phyla found in soil (**Table 1**). Unless specified otherwise, the strains were obtained from the Leibnitz Institute DSMZ-German Collection of Microorganisms and Cell Cultures (DSM Braunschweig, Germany). All strains were routinely grown as pure cultures in 0.5 × Tryptic Soy Broth (TSB) (VWR International, Leuven, Belgium) while shaking at 280 rpm or on TSB agar plates at 30°C. For the incubation of the synthetic community, we used 0.1 × TSB medium. The synthetic community was incubated in two different experimental set-ups: sand microcosms and shaken batch cultures containing quartz sand. The sand microcosm is based on the porous surface model (Dechesne et al., 2008, 2010; Tecon and Or, 2016) with addition of a layer of quartz sand (≈0.5 g; grains size 100–150 μm) placed on the saturated porous ceramic disk (Ø 2.0 cm). The microcosms were connected to a nutrient reservoir that was placed to generate suction (matric potential head), which thus prescribed partially saturated hydration conditions in the porous media hosting the bacterial community. We used hydration conditions near saturation (-0.5 kPa, ‘wet’) and less saturated conditions (-6 kPa, ‘dry’). To prepare the synthetic community, strains were sampled and resuspended from fresh 0.5 × TSB agar plates in 0.5 × TSB liquid medium. Cell density was adjusted to obtain target optical density at 600 nm (OD_600_) of 0.1, and strains were mixed in equal proportion prior to 100-fold dilution in the same medium. Each microcosm was inoculated with 10 μl of the mixed community suspension. Four replicate microcosms were used for each hydration condition. In another (and anterior) experiment, we grew the synthetic community in triplicate batch cultures as a mixed environment (the community included all species listed in **Table 1** with the exception of *R. etli*). We inoculated flasks containing 25 ml 0.1 × TSB and 2.5 g of quartz sand with 25 μl of the mixed community suspension from pregrown liquid culture and incubated them at room temperature with shaking at 120 rpm. We harvested all bacterial cells from the sand microcosms and extracted community DNA after 6 days of incubation. We sampled the batch cultures after 4 days (bacteria grew faster with shaking), and we separated the quartz sand from the liquid to investigate whether some species preferentially attached to sand particles.

### Nucleic Acids Extraction Procedure

We extracted total nucleic acids (NA) from bacterial cultures grown as batch culture and in unsaturated sand microcosms following the method described by Angel (2012). From the 25 ml batch culture, 500 μl were sampled by pipetting and transferred together with 375 μl phosphate buffer (PB, 120 mM, pH 8) to a 2 ml reaction vial containing the Lysing Matrix E (MP Biomedicals, Solon, OH, United States) for nucleic acid extraction. In addition, we pipetted 500 μl from the bottom of the flask to harvest sand grains into a 2 ml reaction container. The sand phase was washed by adding 1.5 ml phosphate buffered saline (PBS, pH 7.4, Gibco, Paisley, United Kingdom) followed by short mixing by inverting and phase separation by gravity for 1 min. The liquid phase was discarded and the washing was repeated twice with same volume of fresh PBS. The washed sand phase was collected with 375 μl PB and transferred to Lysing Matrix E tubes containing three sizes of beads (MP Biomedicals) to proceed for extraction as described below. The sand phase of each microcosm (approximately 0.5 g) was recovered by pipetting with 375 μl PB and immediately transferred to Lysing Matrix E tubes. For cell lysis, 125 μl of 10% sodium dodecyl sulfate (SDS) solution and 500 μl of Tris-EDTA (TE)-saturated phenol were added to the tubes, and then samples were homogenized for 30 s at a speed of 6.45 m s^-1^ in a bead mile (Bead Ruptor, Omni International, Kennesaw, GA, United States). After centrifugation at maximal speed (16,100 *g*) for 3 min, the aqueous supernatant was transferred to a fresh 2 ml Phase Lock Gel tube (Quantabio, Beverly, MA, United States) and the extraction step was repeated for each lysing tube with fresh buffer. We added 780 μl of phenol/chloroform/isoamylalcohol 25:24:1 to the supernatant, and then, the tubes were vortexed and centrifuged for 3 min at maximal speed. The supernatant was transferred to a fresh tube and mixed with 1 volume of chloroform/isoamylalcohol 24:1. The phases were separated by centrifugation (3 min at maximal speed) and the aqueous supernatant was transferred to DNA low-bind tubes (Eppendorf, Hamburg, Germany). To precipitate NA, we added 1 ml of precipitation solution containing 20% polyethylene glycol 8000 (Alfa Aesar, Karlsruhe, Germany), 2.5 M sodium chloride (Merck, Darmstadt, Germany), and 5 μg of glycogen (Omega Bio-Tek, Norcross, GA, United States), tubes were centrifuged at maximal speed for 1 h at 4°C. The obtained NA pellet was washed with ice-cold 75% ethanol followed by 20 min of centrifugation at maximum speed. The ethanol solution was removed and the tubes were left open at room temperature to evaporate residual ethanol. The NA pellet was dissolved in 50 μl of low TE resuspension buffer (10 mM Tris–HCl, 0.1 mM EDTA, pH 8). We used the DNA obtained from total nucleic acid extraction to quantify species abundance; the co-extracted RNA could be used to assess activity and gene expression levels to be related with, e.g., functioning of the consortium. For the qPCR assays, RNA was removed from the sample by treating a 10 μl aliquot with 40 μg/ml RNase A (Promega, Madison, WI, United States) for 15 min at 37°C in a final reaction volume of 50 μl, followed by column purification (NucleoSpin gDNA Clean-Up, Macherey-Nagel, Düren, Germany) with the following modifications to the manufacturer’s instructions: only one washing step was applied with 700 μl of membrane-washing buffer and elution was done with 20 μl Elution Buffer DE. For the preparation of community DNA standards, genomic DNA was extracted from 5 ml of overnight pure cultures of each species using the same protocol for extraction followed by a column purification step. The DNA concentration was determined with the Qubit dsDNA HS (High Sensitivity) Assay (Thermo Fisher Scientific, Zug, Switzerland) on the plate reader Spark M10 (TECAN, Männedorf, Switzerland). NA extraction from one of the replicate sand microcosms failed to produce sufficient amounts of community DNA for the preamplification step, and therefore, this replicate was not included in the microfluidic qPCR assay. Since no signs of technical failure of that microcosm were observed during the incubation period, we surmise that the NA were accidentally lost during the nucleic acid extraction procedure.

### Design of Species-Specific Primers for Quantitative PCR

Universal and species-specific oligonucleotide primers (listed in **Table 2**) were used to trace the species in the community by qPCR. For the PCR preamplification step, we used universal primers 27F and 1492R as described before by Lane (1991), which target conserved regions of the 16S rRNA gene in all bacterial species. The specific primers were designed according to the qPCR guidelines proposed by the producer (Sigma–Aldrich, Buchs, Switzerland). The target sequence for the reverse primer was located 78–165 nucleotides upstream of the target sequence of the forward primer to produce amplicons suitable for qPCR assay. All primers are 18–20 nucleotides long, the melting temperature of the various primer pairs is within 2°C, and GC ratio ranges from 44 to 61%. Primer pairs were designed to be run under similar PCR conditions. Sequences for primer design were obtained from the SILVA rRNA database project^1^ (Pruesse et al., 2007). Primer sequences for PCR reactions were designed based on comparison of highly variable regions from a multisequence alignment of the 16S rRNA genes from all species with BioEdit (Hall, 1999). The specificity of the primers was evaluated *in silico* against the latest sequence collections of established 16S sequence databases of bacterial genes using probeCheck (Loy et al., 2008), as well as experimentally using a mix of all species genomic DNA isolated from pure cultures. Experimentally, we did not observe cross-amplification among target species in mock communities of combined purified bacterial DNA of all individual species excluding the target organism for the 10 species used in batch cultures. Analysis of the primers melting curves showed high primer specificity giving a single distinct peak characteristic for each primer pair (Supplementary Figure 1). Relative efficiency of the target and normalizer were compared in parallel qPCR reactions with the same template (Supplementary Figure 2).

**Table 2:** Primers used in this study.

Name	Sequence (5′ to 3′)	Tm (°C)	CG Content (%)	Length (nt)	Target pos. 16S	Reference
**Species-specific primer pairs**	
A_chlo F	CAGCTTGCTGGTGGATTA	60.5	50.0	18	79–183	This study
A_chlo R	CACCATGCGATGATCAGT	61.3	50.0	18		This study
B_subt F	GACAGATGGGAGCTTGCT	61.1	55.6	18	68–201	This study
B_subt R	TGTAAGTGGTAGCCGAAGC	61.0	52.6	19		This study
B_xeno F	AATACATCGGAACGTGTCCT	61.3	45.0	20	118–283	This study
B_xeno R	TCCTCTCAGACCAGCTACAG	60.3	55.0	20		This study
E_coli F	GAAGCTTGCTTCTTTGCTG	61.0	47.4	19	78–177	This study
E_coli R	TTGGTCTTGCGACGTTATG	62.8	47.4	19		This study
M_lute F	GACATGTTCCCGATCGCC	67.1	61.1	18	992–1126	This study
M_lute R	CCACCATTACGTGCTGGC	65.7	61.1	18		This study
P_prot F	GTACTTGTACCTGGTGGCG	61.2	57.9	19	79–157	This study
P_prot R	GTATTAGCGCCCGTTTCC	62.4	55.6	18		This study
P_sabi F	GAGTTATGATGGAGCTTGCT	59.0	45.0	20	68–180	This study
P_sabi R	GGTATGCACCAGAAGGTCTT	61.1	50.0	20		This study
P_stut F	CTTGCTCCATGATTCAGC	60.1	50.0	18	82–165	This study
P_stut R	ACGTATGCGGTATTAGCGT	60.2	47.4	19		This study
R_etli F	CGCAGGGAAACTTGTGCTAA	65.4	50.0	20	152–270	This study
R_etli R	CTATGGATCGTCGCCTTGG	65.9	57.9	19		This study
S_viol F	GAACGATGAACCACTTCGGTG	65.3	50.0	20	63–179	This study
S_viol R	GATGCCTGCGAGGGTCAGTA	66.2	57.9	19		This study
X_auto F	GATCTACCCAATGGTACGG	59.5	52.6	19	128–209	This study
X_auto R	GTTCATCCAATGGCGATA	60.0	44.4	18		This study
**Universal primer pairs**						
27F	AGAGTTTGATCCTGGCTCAG	61.5	50	20		Lane, 1991
1492R	CGGTTACCTTGTTACGACTT	58.7	45	20		Lane, 1991
534R	ATTACCGCGGCTGCTGG	67.7	64.7	17		Lane, 1991
1099F mod	AACGAGCGCAACCCT	61.2	60	15		modified from Dyksterhouse et al., 1995
1407R mod	GACGGGCGGTGTGTA	60.9	66.7	15		modified from Lane et al., 1985

### Microfluidic Quantitative PCR Assay

**FIGURE 2:**
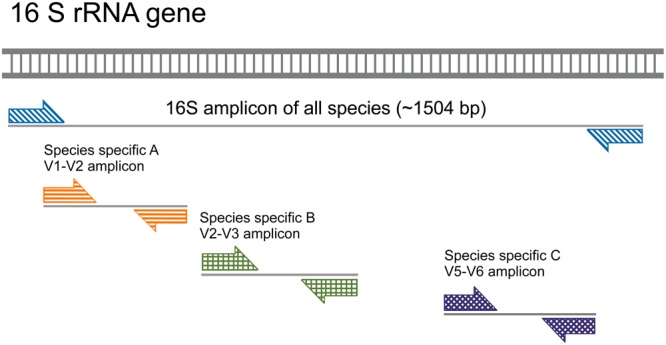
Nested qPCR approach for species-specific amplification of 16S rRNA gene. Sample preparation for qPCR includes a preamplification step with the bacterial universal primer pairs 27F and 1492R in order to increase the number of 16S rRNA gene copies. The amplified 1,504 bp region of the 16S rRNA gene encompasses variable regions that are amplified by species-specific primer pairs in a subsequent (nested) PCR reaction (exemplified here as species A, B, and C and their respective amplicons).

We have used a nested PCR strategy to quantify 16S rRNA gene copy numbers. First, we preamplified the 16S rRNA genes with universal primers 27F and 1492R (**Figure 2** and **Table 2**). This step guaranteed sufficient copies of the target rRNA gene for efficient detection by high-throughput microfluidic qPCR reactions in nanoliter aliquots. A minimum of 800 copies of a target DNA sequence per μL is recommended by the manufacturer (Fluidigm Corporation, San Francisco, CA, United States). The preamplification PCR mixture contained 10 μl of GoTaq G2 Colorless Master Mix (Promega, Dübendorf, Switzerland), 200 nM of each primer, and 4 μl of community DNA template (ranging 2–100 ng) in a final volume of 20 μl. Amplification was performed in a SimpliAmp thermal cycler (Applied Biosystems, Singapore) using the following conditions: initial denaturation at 95°C for 10 min, followed by either 15 cycles or 18 cycles of denaturation at 95°C for 30 s, annealing at 59°C for 30 s, elongation at 72°C for 90 s, followed by a final elongation step at 72°C for 10 min. A total of 5 μl of the PCR product were treated with 10 U of Exonuclease I (Thermo Fisher Scientific, Waltham, MA, United States) in a final volume of 5.5 μl at 37°C for 15 min to eliminate preamplification primers, as recommended in the Fluidigm protocol (‘Eva Green DNA Binding Dye for Gene Expression with the 48 × 48 Dynamic Array IFCs – Advanced Development Protocol’ Version 100–1208 B, free online resource), followed by enzyme inactivation at 85°C for 15 min.

Quantitative PCR was carried out using a Fluidigm Dynamic Array 48 × 48. The exonuclease-treated DNA was diluted 5 times with DNA suspension buffer (10 mM Tris–HCl, 1.0 mM EDTA, pH 8), and then 2 μl of the dilution were added to 4 μl of sample mix containing 1× HOT FIRE Pol EvaGreen qPCR Mix Plus (ROX) (Solis Biodyne, Tartu, Estonia) 1× DNA Binding Dye Sample Loading Reagent (Fluidigm) in water added to the final volume. The assay mix was prepared following the manufacturer’s instructions. Briefly, a master mix was prepared for each primer pair, containing 20 μM of both forward and reverse primer. In a final volume of 6 μl, we combined 2.7 μl of primers mix with 3 μl of 2 × Assay Loading Reagent (Fluidigm Corporation) and 0.3 μl of DNA suspension buffer. The master mix was prepared for each of the primer pairs targeting species-specific regions of the preamplified 16S rRNA gene, and a universal primer sets targeting regions that are conserved among all species (**Table 1**). We have used a reaction without DNA template as negative control. For positive control and calibration, a serial dilution with known DNA concentration from a mixture of genomic DNAs (obtained from pure cultures) of all species was prepared, treated in the exact same manner as the samples and run on the same Fluidigm Dynamic Array. All samples and calibration reactions were run in four technical replicates with the following standard real-time PCR program provided by the manufacturer: UNG reaction at 50°C for 120 s, Hot Start 95°C for 10 min, followed by 40 cycles of 95°C for 15 s and 60°C for 60 s in the final-step melt curves are produced by a temperature increase of 1°C per 3 s from 60 to 95°C.

Considering two contrasting environments, well-mixed batch cultures containing sand (both liquid and sand phase, three replicates each) and partially saturated sand microcosms with wetter or drier conditions (four replicates each), resulting in 14 samples. One replicate sand microcosm incubated under ‘wet’ conditions yielded extremely low amount of DNA that could not be satisfactorily preamplified; for this reason, it was not loaded in the 48 × 48 Fluidigm chip. To these samples we added the respective starting community together with a negative control and a 8-point calibration standard prepared as fivefold dilution series. To compare the effect of preamplification conditions, we compared 15 and 18 amplification cycles for all samples including the standards for the calibration on one chip (a total of 48 DNA samples).

### Data Analysis

For conventional real-time PCR, we used the 7500 software v2.0.6 (Applied Biosystems, Foster City, CA, United States) to analyze generated cycle threshold (*C*t) values (also known as quantification cycle, *C*q). Microfluidic quantitative PCR assays were evaluated with the Fluidigm Real-Time PCR Analysis software Version 4.1.3 (Fluidigm Corporation). Quality threshold is defined by the evolution software of the manufacturer to measure the “quality” of each amplification curve. Comparison of each amplification curve to an ideal exponential curve gives a Quality Score between 0 and 1, where 0 is a flat line and 1 is a perfect sigmoid. We used the default setting with an arbitrary cutoff value of 0.65 for the Quality Threshold in the Real-Time PCR Analysis software.

To present the real-time PCR assessment of community composition, we used the comparative *C*t method (2^-ΔΔC_t_^ method) proposed by Livak and Schmittgen (2001) and Schmittgen and Livak (2008). We assumed that the total amount of bacteria in each microcosm was represented by the universal primer pair that was selected as internal control. The ΔΔ*C*t method requires that the amplification efficiencies of normalizer and target gene are similar. In order to compare efficiencies of normalizer and each specific species, we calculated the Δ*C*t values as *C*t_normalizer_-*C*t_target_ where the universal primer set I served as normalizer. The relative efficiency plot (Supplementary Figure 2) compares performance of each individual primer pair compared to the universal primer pair that was selected as internal control (**Table 2**) and verifies that it is consistent across a range of template concentrations. The relative efficiency plot shows a nearly flat line with a slope in the range of -0.0001 to -0.0218 (Supplementary Table 1) suggesting highly similar efficiency with community DNA input ranging from 0.01 to 100 ng. These results inspire confidence in employing the ΔΔ*C*t method in which a slope of <0.1 is considered acceptable. The melt curve analysis that exhibits high level of primer specificity with a single distinct peak characteristic for each primer pair was observed for all dilutions (Supplementary Figure 1).

**FIGURE 3:**
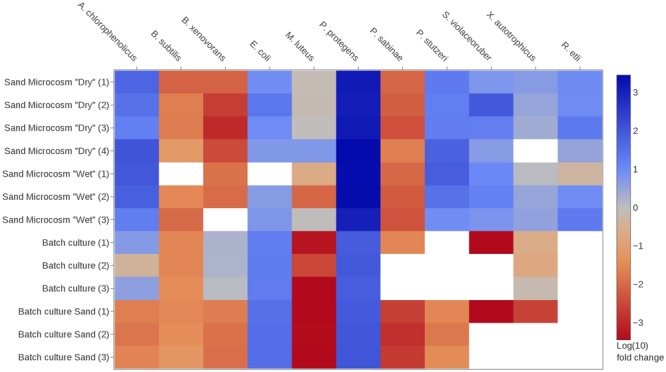
Changes in species relative abundances within the bacterial community under various culture conditions. Community members are described in **Table 1**. Heatmap shows logarithmic fold-change values calculated using the comparative *C*t method (see section “Materials and Methods” for details). Briefly, blue and red colors indicate that the relative abundance of the target species was, respectively, increased or decreased in the community after the period of incubation compared to the initial inoculum composition. White color indicates that no signal was detected in the sample for the target species. Sand microcosms were incubated under two partially saturated conditions (depending on the water potential applied to the microcosm, respectively, –0.5 and –6 kPa, i.e., relatively ‘wet’ or ‘dry’ conditions), with four replicates per condition. One ‘wet’ replicate yielded insufficient DNA for preamplification and was not used in the microfluidic qPCR run; therefore, results from three replicates are shown. Results for shaken batch cultures (liquid or sand fraction) are shown in triplicates. The species *Rhizobium etli* was only introduced in the sand microcosms.

To quantify changes in species relative abundances within the final community (DNA samples from microcosms or batch cultures) relative to their abundances in the initial community (suspension of mixed species used as inoculum), we exported data from the Fluidigm software in R to analyze relative species abundance based on the 2^-ΔΔC_t_^ method (**Figure 3**) using the following equation:

$$\begin{array}{c} \mathrm{Fold}\mathrm{change}\;=\;2^{-\Delta \Delta C_{t}} \end{array}$$

With ΔΔCt = ${\mathrm{\Delta Ct}}_{i}^{\mathrm{Final}}$-${\mathrm{\Delta Ct}}_{i}^{\mathrm{Initial}}$ = [Ct_i_ – Ct_univ_]^Final^ – [Ct_i_ – Ct_univ_]^Initial^. The Δ*C*t value of a given species *i* in a community DNA sample was determined by subtracting a reference value: ΔCt_i_ = Species i average Ct value-Universal Primer average Ct value. We used the Universal Primer set 1099F and 1407R optimized to target a 308 bp region of the 16S rRNA gene in all species as internal control to normalize data. The mean *C*t values were used for 2^-ΔΔC_t_^ transformation after normalization with internal control.

**FIGURE 4:**
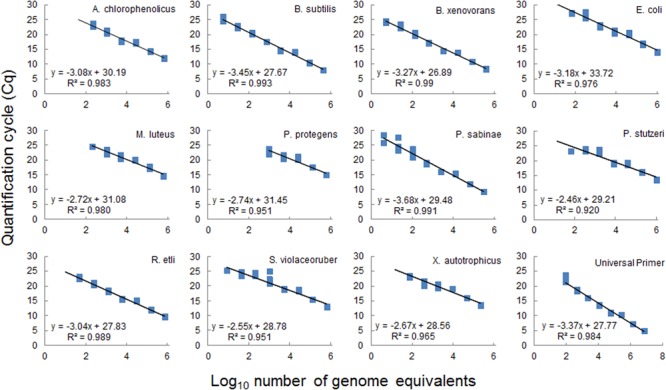
Standard calibration curves for target bacterial species used in the microfluidic assay. Curves were constructed from fivefold serial dilutions of a mixture of purified genomic DNA from eleven individual species (**Table 1**) pooled in equal amounts and run in parallel reaction with each individual species-specific primer pair or with a universal primer pair (**Table 2**). Based on genomic DNA concentrations in the stock solution and on the genome size the number of genome equivalent copies was calculated (see Supplementary Table 2 for details) and plotted against the cycle threshold for each species-specific qPCR assay. Data evaluation was performed with the Fluidigm software from four technical replicates per dilution. Data points for each replicate are displayed as blue square. Equations of fitted linear regression lines and *R*^2^ values are shown, calculated from average *Cq* values.

The absolute abundance of each species was calculated by transformation of the data via a standard curve (**Figure 4**). Genomic DNAs extracted from pure cultures of all bacterial species were combined in equal proportions and used to prepare a series of fivefold dilutions for generating the standard curve. We used the calibration view from the Fluidigm software to plot *C*t values versus the amount of template DNA (log genome equivalents, Supplementary Table 2), and we then fitted a standard curve by linear regression. The goodness of fit (*R*^2^) and the amplification efficiency (derived from the slope) were calculated for each curve (**Figure 4** and Supplementary Table 3). The calibration curves were used to calculate the number of species genome equivalents in the community DNA obtained from a given sample. Measurement values below the theoretical limit of detection (<1 genome in DNA template) were not reported (Bustin et al., 2009). DNA concentration measured fluorometrically using the Qubit dsDNA HS (High Sensitivity) assay kit after extraction of nucleic acid from entire microcosms was employed to conclude size of each microbial consortium and was taken into account for comparison of community composition and abundance of individual species. We removed 0.5 ml aliquot from the 25 ml batch cultures for nucleic acid extraction. To conclude absolute abundances for each microcosms and for the entire batch cultures, the obtained values were calculated for the total volume of the microcosm and per ml of the batch culture, respectively.

## Results

### Changes in Relative Species Abundance under Different Environmental Conditions

We hypothesized that contrasting environmental conditions would alter the composition of the representative soil bacterial community (**Table 1**), and that these changes could be quantified (at the species level) using qPCR (**Figure 1**). We first assessed how the relative abundance of a given species in the community varied from its initial inoculum to its final composition (extracted sample) using comparative *C*t analysis (see section “Materials and Methods”). Typically, this method calculates whether a given species is more or less abundant in the community at the sampling time relative to the beginning of the incubation period. In other words, it reveals whether a species experienced competitive advantage or disadvantage over other community members under the given growth conditions. **Figure 3** depicts results of comparative *C*t analysis in the experiments. Growth in hydration-controlled sand microcosms (spatially structured environment) or in batch cultures containing sand (well-mixed environment) had different and sometimes opposite effects on the final relative abundance of species in the community. *Pseudomonas protegens* clearly dominated all sand microcosms (with up to a 1,000-fold change increase in relative abundance). Certain species (*P. stutzeri, Arthrobacter chlorophenolicus*, and *Streptomyces violaceoruber*) became relatively more abundant in all sand microcosms, but declined or were not detected at all when the community was grown in batch cultures (**Figure 3**). *Escherichia coli* and *P. protegens* clearly dominated all batch cultures with up to 100-fold increase in relative abundance, but changes in sand microcosms were more variable for *E. coli*. Several species appeared to be unaffected or to decline in the community regardless of the environmental conditions (*Burkholderia xenovorans, Bacillus subtilis, Paenibacillus sabinae*, and *Micrococcus luteus*). Statistical comparisons of sand microcosms versus liquid cultures showed significant differences in fold change in relative abundance for several species (*p*-value < 0.05 with a two-tailed *t*-test), and these differences were more pronounced between ‘dry’ microcosms and batch cultures than between ‘wet’ microcosms and batch (8 and 3 species with *p*-values < 0.05, respectively). The growth medium was the same in all experiments (0.1 × TSB), indicating that factors other than nutrients composition acted on the relative species fitness. Relatively few differences were observed between results obtained with the liquid phase and with the sand phase in batch cultures (**Figure 3**). However, *B. xenovorans* showed significant relative decrease in the sand fraction compared to liquid (*p*-value < 0.01), while *P. stutzeri* and *P. sabinae* were detected in the liquid fraction but hardly or not at all in the sand fraction. In sand microcosms, wetter or drier conditions (imposed by water potential) had limited effects, and analysis showed no significant differences in fold change for species in ‘wet’ and ‘dry’ microcosms.

### Absolute Species Abundance

**FIGURE 5:**
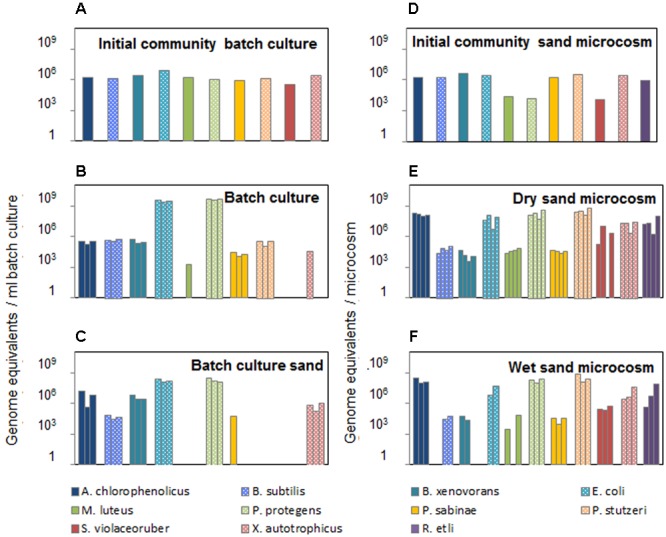
Absolute species abundance in the bacterial community after incubation under contrasted conditions. Species abundance is expressed as number of genome equivalents (calculated from calibration curves) per milliliter of liquid batch culture **(A–C)** or per microcosm **(D–F)**. **(A, D)** Species abundances in the initial community used to inoculate batch cultures in shaken flasks or sand microcosms. One additional soil bacterial species (*R. etli*) was added to the community inoculated into sand microcosms **(D)**. Bacterial community composition was assessed in triplicate batch cultures after 4 days of incubation at 23°C, both in the liquid phase **(B)** and in the sand fraction **(C)**. Four replicate soil microcosms were incubated for 6 days at 23°C with two controlled hydration levels (‘dry’ at –6 kPa or ‘wet’ at –0.5 kPa, respectively, **E** and **F**). One replicate ‘wet’ sand microcosm yielded insufficient DNA for preamplification and was not used in the microfluidic qPCR run; therefore, results from three replicates are shown **(F)**.

To estimate the absolute abundance of each species in a multispecies community under different experimental conditions, we generated standard calibration curves from a mix of purified genomic DNAs with known concentration (**Figure 4**). All strains in the community are well characterized with known sequenced genome information; required values of genome equivalents and number of 16S gene copies were determined for each species in the calibration assays (Supplementary Table 2). The standard curves were obtained within a similar range from 10 to 10^6^ equivalent genomes in a dilution series (**Figure 4**). The resulting curves were linear with *R*^2^ in the range 0.92–0.99 for all species. The curves slopes were within a range from -3.7 to -2.5, which translates into a range of amplification factors and efficiencies for the various species-specific reactions (Supplementary Table 3). Variations in amplification factors influence the final copy number of the target genes obtained after thermocycling, and this effect increases with increasing PCR cycles. However, this effect is canceled out in the calculation of species absolute abundances, since the genomic DNA of a given species, which is present both in the sample and in the calibration mix, is submitted to the same amplification biases throughout the assay. Calibration curves were used to calculate species absolute abundances in the sand microcosms and batch cultures (**Figure 5**). The bacterial community growing in sand microcosms shows species abundance ranging from 3.29 × 10^3^ genome equivalents (*M. luteus*) to 8.85 × 10^8^ genome equivalents (*P. stutzeri*) and, per ml batch culture, from 1.75 × 10^3^ equivalents (*M. luteus*) to 5.55 × 10^9^ (*P. protegens*) genome equivalents.

## Discussion

Studies of bacterial communities from diverse environments often rely on high-throughput sequencing technology that generates a snapshot of the community composition by providing relative abundance at taxon level (Widder et al., 2016). Ecological interpretations of community functioning, dynamics, and productivity often require more detailed information, including species-level resolution of community members to identify keystone species and quantification of absolute abundances to evaluate community dynamics and compare community composition from different samples (Philippot et al., 2009; Berry and Widder, 2014). A recent study has reported progress in estimating the absolute abundance of soil bacteria using sequencing methods (Smets et al., 2016), however, the authors note that accuracy is not yet satisfactory and that qPCR with taxon-specific primers remains the most suitable method to determine absolute abundances. So-called ‘next generation qPCR’ is now available on a variety of microfluidic PCR platforms (Devonshire et al., 2013, see section “Materials and Methods”). Microfluidic qPCR uses nanoliter volumes for the thermal cycling reactions, enabling thousands of individual qPCR assays on a single chip, relies on automatic distribution of samples and assays that limits pipetting to a minimum, and produces quantitative data that are highly correlated to data obtained with conventional microliter qPCR (Spurgeon et al., 2008). In this context, we developed a microfluidic-based qPCR assay (**Figure 1**) as an effective high-throughput method for resolving changes in the composition of a multispecies community at various taxonomic levels, while measuring relative and absolute abundances at species level, and detecting changes in community composition following exposure to different environmental conditions. In particular, the method could be used for hypothesis testing and evaluate predictions pertaining to changes in synthetic or representative bacterial communities across a range of habitats. Additionally, the method may be used for detection of so-called ‘keystone’ or ‘tracer’ species within natural communities in a variety of environments [such as the monitoring of *B. xenovorans* LB400 used in the bioremediation of soils contaminated with polychlorinated biphenyls (Norini et al., 2013) or the detection of multiple enteric pathogens in fecal and water samples (Ishii et al., 2013)]. To evaluate the method, we have used a well-characterized community composed of 10–11 bacterial species representing bacterial phyla commonly isolated from soil environments (**Table 1**). The community was grown under partially saturated conditions in sand microcosms and within shaken batch cultures to provide contrasting environments. For extraction of nucleic acid, we adopted a protocol for simultaneous isolation of DNA (to assess community composition) and RNA [to analyze functional activities (Angel et al., 2013)]. PCR inhibition by co-extracted compounds is a main challenge when dealing with environmental samples like soil (PCR inhibition by humic acids) or clinical samples as tissue or blood (PCR inhibitors like IgG, hemoglobin, and lactoferrin). In order to avoid PCR inhibition through components of the medium the cells are grown in, we use a system which is based on a previously sterilized sand microcosm inoculated with a synthetic bacterial community. The used extraction method uses phenol–chloroform which could possibly lead to carry over of phenol residuals that are known PCR inhibitors. Therefore, we introduced an additional cleanup step which includes RNA digestion and column purification. The resulting purified DNA is used in downstream reactions. A preamplification step was included in the workflow to increase the target, as cultures from dry sand phase typically return little amount of DNA for subsequent analysis. Since previous studies reported on non-uniform target enrichment and unsuccessful preamplification steps (Korenková et al., 2015; Wang et al., 2015), we optimized preamplification with design of a nested PCR using the 16S rRNA gene as target (**Figure 2**). This nested PCR approach allowed us to keep the species-specific primer pairs in separate singleplex reactions and circumvent cross-amplification bias and chimera formation in a multiplex assay with a mix of all 11 primer pairs with high sequence similarity (von Wintzingerode et al., 1997). We compared the amplification rate in a reaction with species-specific primers from the same assay using the universal primer set and found highly similar results indicating that the universal primer pair has similar reaction efficiency than the species-specific primer pairs. This finding is supported by the relative efficiency plot for each individual standard calibration curve (Supplementary Figure 1 and Table 1) that allows a direct comparison of efficiencies for both the normalizer and the target gene. The obtained plots show all a flat line with slope <0.1 (Supplementary Table 1) that indicates acceptable efficiency across relevant input concentrations, a requirement for the application of the ΔΔ*C*t method (Schmittgen and Livak, 2008). PCR selection due to binding site variation in a single position of the universal primer pair and difference in GC content increases with the number of amplification cycles and can lead to an over-amplification of certain target species (Suzuki and Giovannoni, 1996; Polz and Cavanaugh, 1998). Since we did not use degenerated primers in this study, the universal primers possess a single-position mismatch for the *E. coli, P. protegens*, and *P. stutzeri* target region in the 16S rRNA gene, but despite the mismatch all three species were easily detected in our Fluidigm assay, often producing a strong signal (**Figure 3**). To minimize PCR effects during the preamplification step, we have used the data set obtained with a lower number of cycles (15 cycles) as proposed by the manufacturer. However, we note that results obtained with 18 instead of 15 cycles of DNA preamplification are very similar (Supplementary Figure 4), and thus it would be acceptable to use 18 cycles when only low amounts of DNA starting material are available. To ensure that even low abundant species are captured in the assay, which would otherwise fail to pass the detection limit, we recommend to generate a range of community DNA concentrations and run replicated assays with low load, high load, and moderate load for a more accurate measurement on the same chip, especially for samples with a broad range of species abundance. Advantage of the Fluidigm 48 × 48 chip assay is that the standard calibration can be run in parallel with the samples, which helps to avoid interassay imprecisions. An optimal standard curve should cover the effective assay range. Generally, a series of fivefold dilutions that provides a 5-point standard calibration curve is sufficient.

The method was used to track changes in bacterial community composition grown in structured environments with different saturation levels or in a mixed aquatic environment. The synthetic community used in this study showed a strong response to the habitat structure, with higher diversity in presence of solid surfaces and connectivity of aquatic habitats determined by hydration levels (**Figures 3, 5**). In batch cultures, two species (*M. luteus* and *S. violaceoruber*) showed very low *C*t values below theoretical detection limit that did not permit absolute quantification, whereas in the heterogeneous sand microcosms, we were able to detect and quantify all species. The resulting trends in bacterial abundance suggested a link to specific ecological strategies. While a homogenous environment promoted abundance of rapidly growing copiotrophic bacteria such as *E. coli* and *P. protegens* (Leadbetter and Poindexter, 1985; Boutte and Crosson, 2013; Lladó and Baldrian, 2017), the slower growing species (*M. luteus* and *S. violaceoruber*) dropped below (theoretical) detection limit (**Figures 3, 5**). In contrast, the structured microcosm environment promoted slow growing species (oligotrophs) and enabled them to coexist with copiotrophic strains. Moreover, there is a tendency for motile bacterial species to increase in abundance in an aqueous environment compared to a more porous habitat with a water configuration that limits cell distribution (Dechesne et al., 2010). The community composition in all three replicated batch cultures showed high similarity of species relative and absolute abundances (**Figures 3, 5**), which indicated that the community assembly was highly reproducible under those conditions. Incubation in partially saturated sand microcosms led to slightly more variance in community composition. Overall, this first insight suggested that a structured environment supports a broader range of bacterial species with differences in physiology, metabolic potential (to use the given nutrient resources), and ability to move under sufficient hydration conditions.

We were able to detect individual species from different phyla as well as closely relate species from the same genus in our assay, using the well-studied 16S rRNA gene. However, a drawback associated with using the 16S rRNA gene is the potential for high similarity among closely related species with primer target sites that differ in only a few bases, which limits the design of unique primers or imposes the use of phylogenetically distant members in a synthetic community. We thus would recommend using primer pairs that target 16S and 23S rRNA genes to ensure primer specificity especially for natural communities, e.g., from environment or for clinical samples.

We applied the described method to a synthetic microbial ecosystem comprising of a representative bacterial community grown in well-controlled and characterized microcosms under defined hydration conditions. This simple synthetic microbial ecosystem allowed for systematic control of abiotic factors, while keeping all other factors constant, which makes the system highly suitable for testing hypotheses pertaining to biodiversity theory (e.g., Bell et al., 2005; Langenheder et al., 2012; Fetzer et al., 2015). While in the presented study the synthetic community was inoculated into sterile environments, the method could be used for non-sterile habitats to study effects of biotic factors (e.g., the influence of the indigenous microbial community) on the abundance of the synthetic (tracer) bacterial community. Moreover, the sets of primers developed here may be useful for studying natural soil community DNA samples (given that our assay targeted common soil species). Although we did not explore this possibility in the current study, the co-extraction of RNA as described in the protocol could be used to link species abundance with community functioning and metabolic activities through quantification of functional genes that provide information on the genetic potential of community members to catalyze certain processes (Peng et al., 2017). Thus, the new approach is suitable to identify general patterns, processes, and functions from a designed consortium or synthetic community that also occur and operate in more complicated ecosystems and can find application to detect members of interest in indigenous communities found soil or other environment harboring microbial life, or even in non-microbial ecosystems (Prosser et al., 2007; O’Malley et al., 2015).

Finally, the approach described in this study could be adjusted to quantify target indicator species in natural environments. Such indicator species could be, for example: *Rhizobium* spp. linked to terrestrial ecosystem health (van Bruggen and Semenov, 2000); in aquatic environments *Aeromonas* spp. and *Pseudomonas* spp. as indicators of bacterial regrowth in water distribution systems (Ribas et al., 2000); to track fecal water pollution (Nebra et al., 2003); in the clinical field for fast quantification of marker for the microbiome (Berry et al., 2012). The proposed method could replace previous low-throughput real-time PCR detection systems and give a better understanding of species–species interactions within bacterial communities that need quantitative data on species abundance rather than solely presence or absence (Berry and Widder, 2014). For natural bacterial communities, the proposed workflow could be adapted by combining phyla or taxon-level qPCR quantification with barcoded primers targeting the ribosomal DNA followed by transfer of the PCR products from the chip to a sequencing reaction to resolve community composition at species and OTU level (Hermann-Bank et al., 2013).

## Author Contributions

HK, RT, and DO conceived the study and wrote the manuscript. HK performed the experiments and analyzed the data.

## Conflict of Interest Statement

The authors declare that the research was conducted in the absence of any commercial or financial relationships that could be construed as a potential conflict of interest. The reviewer SS and handling Editor declared their shared affiliation.

## Footnotes

1 https://www.arb-silva.de/
