# Supplementary material for: Resolving Species Level Changes in a Representative Soil Bacterial Community Using Microfluidic Quantitative PCR
Source: Front Microbiol. 2017 Oct 25;8:2017. doi: 10.3389/fmicb.2017.02017 (PMC5661172; doi:10.3389/fmicb.2017.02017)
Supplement: Supplementary file 1 [file Presentation_1.PDF]

Supplementary Material

Resolving species level changes in a representative soil bacterial community using microfluidic quantitative PCR

Hannah Kleyer [1*], Robin Tecon[1], Dani Or[1]

[1]Soil and Terrestrial Environmental Physics, Department of Environmental Systems Science, ETH Zürich, Universitätstrasse 16, 8092 Zürich, Switzerland

*** Correspondence:**
Hannah Kleyer
hannah.kleyer@usys.ethz.ch

Supplementary Figure 1.

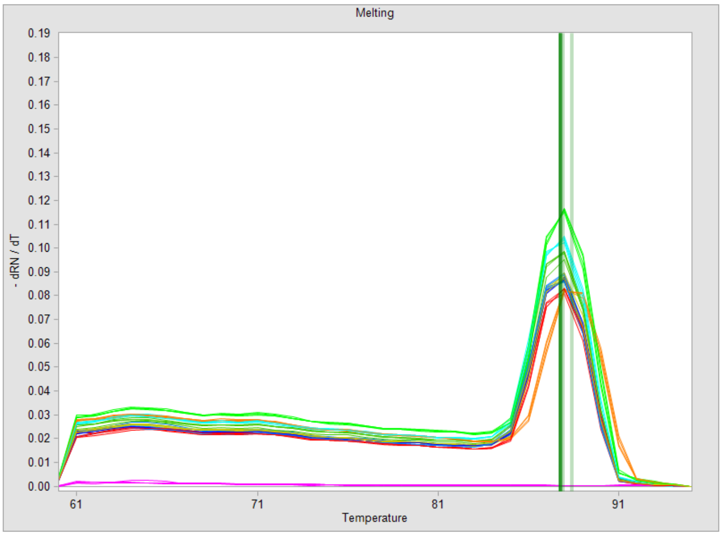

Quantitative real-time PCR analysis of melting curve for species specific primer pair targeting *B. subtilis* in the calibration curve dilution series. Melt curve analysis for species specific primers as shown here exemplarily for a dilution series of *B. subtilis*. Lower dilution levels show high specific melting curve with one single distinct peak at 88 °C. The no template control is shown in pink, no signal was detected in the negative control.

This is a provisional file, not the final typeset article

Supplementary Figure 2.

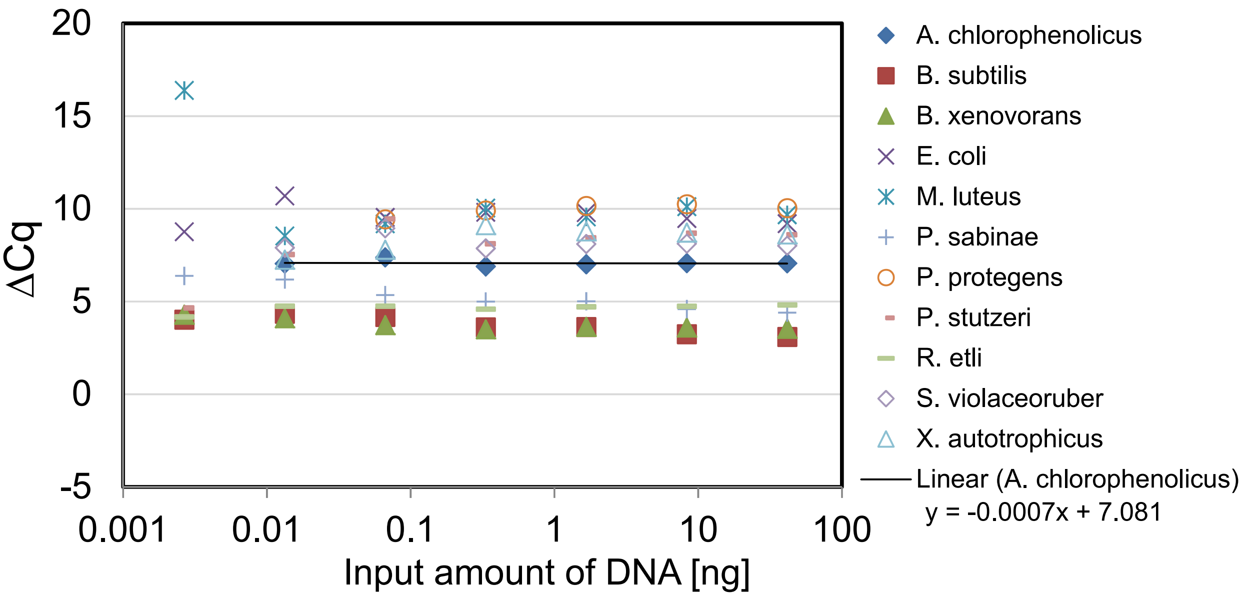

Relative efficiency plot for each individual standard calibration curve. The ΔΔCt method requires comparable efficiencies for both the normalizer and target gene. In order to compare efficiencies of normalizer and each specific species we calculated the ΔCt values as $Ct_{normalizer} – Ct_{target}$ where the universal primer set I served as normalizer. When plotted against serial dilution with known amount of DNA, a slope is obtained (Supplementary Table 2). While a perfectly flat line (slope = 0) indicates identical efficiency across all input concentrations, a slope of <0.1 is generally considered acceptable when employing the ΔΔCt method (Schmittgen and Livak, 2008).

Supplementary Figure 3.

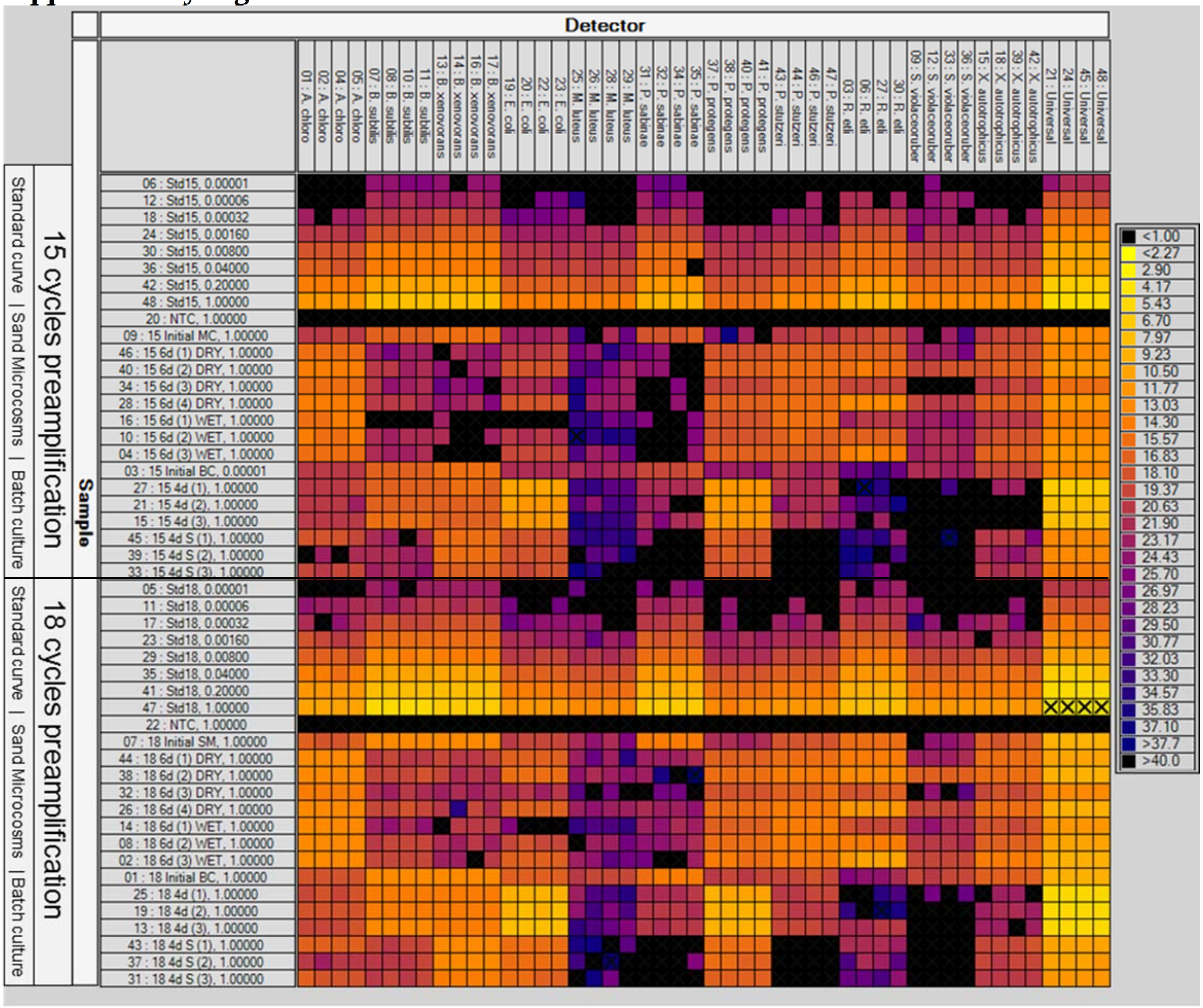

Heatmap generated by the Fluidigm Real-Time PCR Analysis program after thermal cycling of the 48x48 dynamic array. Each square represents one reaction chamber on the microfluidic chip (2,304 reactions in total). Square color indicates Cq value according to the color scheme shown on the right side. Black color means that no target was detected. Rows correspond to the calibration standards and to the community DNA samples, preamplified with 15 (top rows) or 18 (bottom rows) PCR cycles (see legend on the left side). The two entirely black rows correspond to ‘no DNA template’ negative controls. Columns correspond to the species-specific and universal assays (based on primers specificity), each assay being run in four technical replicates. Values that did not pass the default quality threshold of 0.65 (see Material and Methods) are marked with the symbol X and were not analyzed. The primer pair designed to detect *R. etli* produced a weak signal (below theoretical detection limit) in some of the DNA samples from batch cultures, even though *R. etli* was not present in batch. This could be an indication that limited unspecific DNA amplification took place, albeit negligible for quantification of absolute abundance

This is a provisional file, not the final typeset article

Supplementary Figure 4.

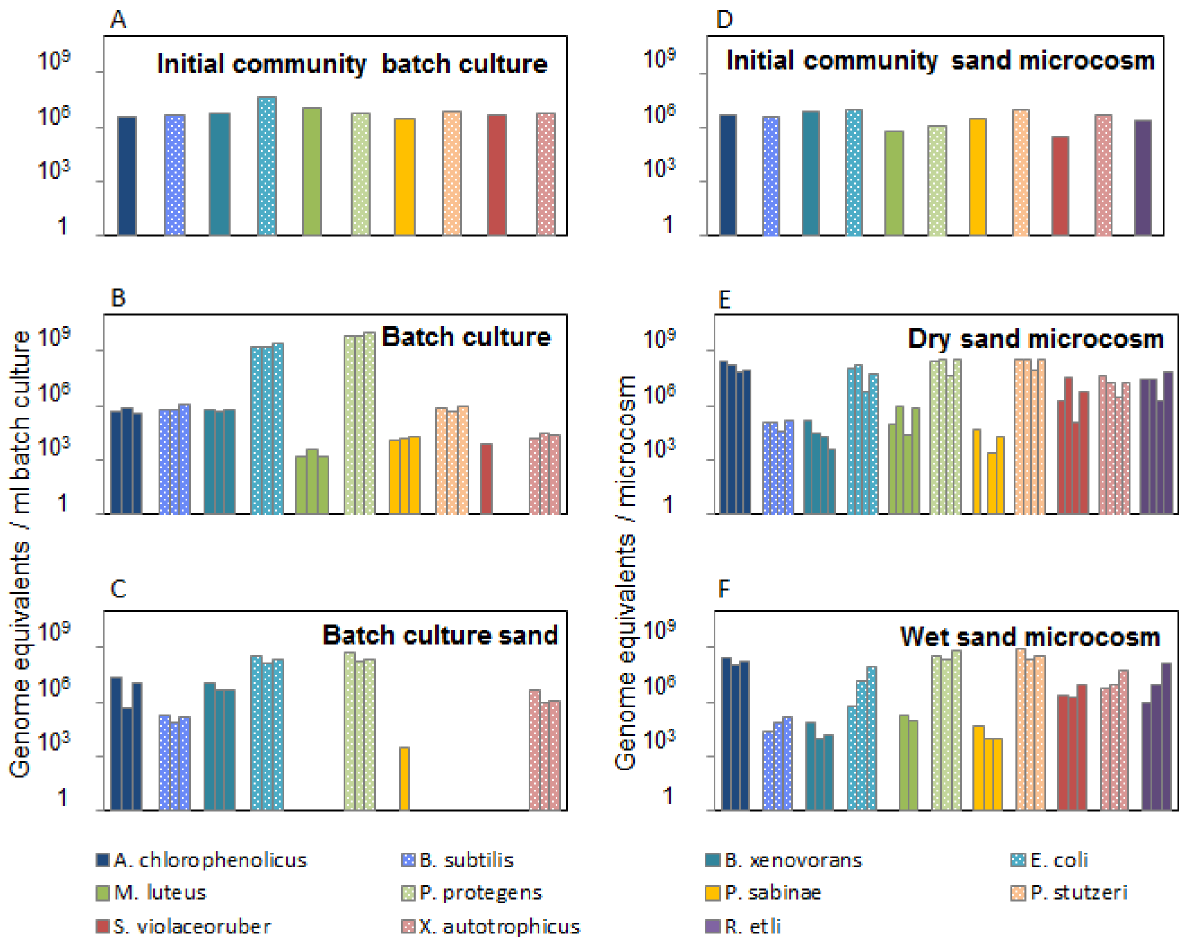

Absolute species abundance in the bacterial community after incubation under contrasted conditions. Results were produced using a preamplification step of 18 PCR cycles (instead of the recommended 15 cycles as in Fig. 5). This was done to test the effects of additional preamplification cycles and to enhance potential detection for low abundant species. Species abundance is expressed as number of genome equivalents (calculated from calibration curves) per milliliter of liquid batch culture (A-C) or per microcosm (D-F). A, D. Species abundances in the initial community used to inoculate batch cultures in shaken flasks or sand microcosms. One additional soil bacterial species (R. etli) was added to the community inoculated into sand microcosms (D). Bacterial community composition was assessed in triplicate batch cultures after 4 days of incubation at 23 °C, both in the liquid phase (B) and in the sand fraction (C). Four replicate soil microcosms were incubated for 6 days at 23 °C with two controlled hydration levels (‘dry’ at –6 kPa or ‘wet at –0.5 kPa, respectively E and F). One replicate ‘wet’ sand microcosm yielded insufficient DNA for preamplification and was not used in the microfluidic qPCR run, therefore results from three replicates are shown (F).

Supplementary Table 1.

Species	Slope
A. chlorophenolicus	-0.0007
B. subtilis	-0.0218
B. xenovorans	-0.0001
E. coli	-0.0127
M. luteus	-0.0086
P. protegens	-0.0047
P. sabinae	-0.017
P. stutzeri	-0.0052
R. etli	-0.0007
S. violaceoruber	-0.0049
X. autotrophicus	-0.0099

In order to compare efficiencies of normalizer and each specific species we calculated the ΔCt values as $Ct_{normalizer} - Ct_{target}$ where the universal primer set I served as normalizer. When plotted against serial dilution with known amount of DNA, a slope is obtained. While a perfectly flat line (slope = 0) indicates identical efficiency across all input concentrations, a slope of <0.1 is generally considered acceptable when employing the ΔΔCt method (Schmittgen and Livak, 2008).

This is a provisional file, not the final typeset article

Supplementary Table 2

Species	Genome size [bp]*	Molar mass [g/mol]	Genome equivalents/ng	16S rRNA gene copies**
A. chlorophenolicus A6	4980870	3.24E+09	1.86E+05	5
B. subtilis 168 trp+	4215606	2.74E+09	2.20E+05	10
B. xenovorans LB400	9731138	6.33E+09	9.52E+04	6
E. coli K-12 MG1655	4641652	3.02E+09	2.00E+05	7
M. luteus DSM20030	2501097	1.63E+09	3.70E+05	2
P. protegens CHA0	6867980	4.46E+09	1.35E+05	5
P. sabinae T27	5270569	3.43E+09	1.76E+05	9
P. stutzeri CMT.9.A	4689946	3.05E+09	1.98E+05	4
R. etli CFN42	6529000	4.24E+09	1.42E+05	3
S. violaceoruber A3(2)	9054847	5.89E+09	1.02E+05	6
X. autotrophicus Py2	5625098	3.66E+09	1.65E+05	2

* Information on genome size obtained from the KEGG GENOME Database - GenomeNet www.genome.jp/kegg/genome.html
** Number of 16S rRNA copies were obtained from rrnDB - the Ribosomal RNA Operon Copy Number Database https://rrndb.umms.med.umich.edu/

Supplementary Table 3.

	15 Cycles Preamplification	18 Cycles Preamplification
A. chlorophenolicus	Amplification factor = 2.11<br>Efficiency = **111.19%**	Amplification factor = 2.02<br>Efficiency = **101.78%**
B. subtilis	Amplification factor = 1.95<br>Efficiency = **94.92%**	Amplification factor = 1.83<br>Efficiency = **83.30%**
B. xenovorans	Amplification factor = 2.02<br>Efficiency = **102.21%**	Amplification factor = 1.93<br>Efficiency = **92.71%**
E. coli	Amplification factor = 2.06<br>Efficiency = **106.28%**	Amplification factor = 1.86<br>Efficiency = **85.70%**
M. luteus	Amplification factor = 2.33<br>Efficiency = **133.16%**	Amplification factor = 2.13<br>Efficiency = **112.75%**
P. sabinae	Amplification factor = 1.87<br>Efficiency = **86.96%**	Amplification factor = 1.85<br>Efficiency = **84.78%**
P. protegens	Amplification factor = 2.32<br>Efficiency = **131.72%**	Amplification factor = 2.30<br>Efficiency = **129.62%**
P. stutzeri	Amplification factor = 2.55<br>Efficiency = **154.98%**	Amplification factor = 2.20<br>Efficiency = **120.02%**
R. etli	Amplification factor = 2.13<br>Efficiency = **113.28%**	Amplification factor = 1.98<br>Efficiency = **98.44%**
S. violaceoruber	Amplification factor = 2.47<br>Efficiency = **146.69%**	Amplification factor = 1.91<br>Efficiency = **90.94%**
X. autotrophicus	Amplification factor = 2.37<br>Efficiency = **136.88%**	Amplification factor = 2.03<br>Efficiency = **102.65%**
Universal Primer	Amplification factor = 1.98<br>Efficiency = **98.03%**	Amplification factor = 1.89<br>Efficiency = **89.24%**

To assess the assay performance the PCR efficiency was determined for each primer pair. Based on the slope of each standard calibration curve the amplification efficiency was calculated with the qPCR Efficiency Calculator online tool provided by Thermo Scientific: https://www.thermofisher.com/ch/en/home/brands/thermo-scientific/molecular-biology/molecular-biology-learning-center/molecular-biology-resource-library/thermo-scientific-web-tools/qpcr-efficiency-calculator.html (last visited 09-2017)

This is a provisional file, not the final typeset article

1 Reference

Schmittgen, T.D., and Livak, K.J. (2008). Analyzing real-time PCR data by the comparative C-T method. *Nat. Protoc.* 3**,** 1101-1108.
